# Draft Genome Sequence of *Sulfitobacter* sp. CB2047, a Member of the *Roseobacter* Clade of Marine Bacteria, Isolated from an *Emiliania huxleyi* Bloom

**DOI:** 10.1128/genomeA.01125-14

**Published:** 2014-11-06

**Authors:** Nana Y. D. Ankrah, Thomas Lane, Charles R. Budinoff, Mary K. Hadden, Alison Buchan

**Affiliations:** a Department of Microbiology, University of Tennessee, Knoxville, Tennessee, USA; b Department of Entomology and Plant Pathology, University of Tennessee, Knoxville, Tennessee, USA; c Graduate Program in Genome Science and Technology, University of Tennessee, Knoxville, Tennessee, USA

## Abstract

We announce the draft genome sequence of *Sulfitobacter* sp. strain CB2047, a marine bacterium of the *Roseobacter* clade, isolated from a phytoplankton bloom. The genome encodes pathways for the catabolism of aromatic compounds as well as transformations of carbon monoxide and sulfur species. The strain also encodes a prophage as well as the gene transfer agent (GTA), both of which are prevalent among members of the *Rhodobacterales* order.

## GENOME ANNOUNCEMENT

Members of the *Roseobacter* lineage of marine bacteria are abundant in the world’s oceans, metabolically versatile, and play important roles in various marine biogeochemical cycles (1). For instance, members of the *Sulfitobacter* genus of the *Roseobacter* clade have been shown to play important roles in sulfur cycling in the ocean, transforming both organic and inorganic forms of the compound (1, 2). Dimethylsulfoniopropionate (DMSP)-producing phytoplankton species, such as the coccolithophorid *Emiliania huxleyi*, are important sources of organic sulfur in marine waters and documented *Roseobacter* niches (1).

*Sulfitobacter* sp. CB2047 was isolated during an induced *Emiliania huxleyi* bloom in Raunefjorden, Norway, by direct plating onto 0.22-µm filtered fjord seawater agar plates supplemented with dimethylsulfoniopropionate (DMSP) as the sole carbon source (3). The genome was sequenced using Illumina technology with an average sequencing coverage of approximately 600×. The genome was assembled into 12 contigs, ranging in size from 18 kb to 2.3 Mb, using CLC Assembly Cell (CLC bio, Cambridge, MA, USA). The genome of CB2047 was annotated using the NCBI Prokaryotic Genome Annotation Pipeline (http://www.ncbi.nlm.nih.gov/genome/annotation_prok/) and the KAAS genome annotation and pathway reconstruction server (4).

The draft genome sequence of *Sulfitobacter* sp. CB2047 is 3,767,790 bp (3.76 Mb) with a G+C content of 60.3%. The genome contains 3,563 coding sequences and 37 predicted tRNAs. Based on 16S rRNA gene sequence similarity, CB2047 is 99.93, 99.93, 98.31, and 100% identical to *Sulfitobacter* strains EE36 (AALV01000001), 3SOLIMAR09 (AXZR00000000), FIGIMAR09 (JEMU00000000), and NAS-14.1 (AALZ01000001), respectively. The genome of CB2047 is lysogenized by a mitomycin-inducible 42 kb prophage (KM233261) that has high sequence homology to other *Sulfitobacter* sp. CB2047 infecting phages: ΦCB2047-A and ΦCB2047-C (5). Genome-wide nucleotide similarity alignments with the ΦCB2047-A and ΦCB2047-C genomes showed that the prophage shares 79 and 74% nucleotide identity, respectively. The genome also possesses the gene transfer agent (GTA) gene cluster, including the diagnostic capsid protein gene, *g5* (KFC25944.1) (6).

*Sulfitobacter* sp. CB2047 harbors several central carbon metabolic pathways, including glycolysis, the tricarboxylic acid cycle (TCA), and the pentose phosphate pathway. Furthermore, pathways for aromatic compound catabolism are present, including the protocatechuate branch of the β-ketoadipate pathway (*pcaGH* [KFC25619.1, KFC25620.1]) and the phenylacetate catabolic pathway (*paaABX* [KFC27335.1, KFC27336.1, KFC27331.1]). Also present are genes for sulfur metabolism, including the DMSP-cleavage enzyme (*dddL* [KFC25711.1]) and the Sox enzyme system (*soxXYZABCD* [KFC25464.1 to KFC25470.1]), which oxidizes thiosulfate to sulfate (7) Carbon monoxide dehydrogenase (*coxSLM* [KFC25996.1 to KFC25998.1]), which mediates the reversible conversion between carbon monoxide and carbon dioxide (8), is present in this genome. Also present is an *N*-acyl-l-homoserine lactone (AHL) synthetase homolog (*luxI* [KFC28110.1]), indicating the strain may be capable of utilizing AHL-based quorum sensing. However, no genes with homology to AHL binding response regulators (*luxR*) were identified. Indeed, the presence of “orphan” *luxI* genes appears common among *Sulfitobacter* species (9). The genome also encodes for a type IV secretion system (*virB1,2,3,4,6,8,9,10* [KFC27915.1 to KFC27903.1]), that may facilitate interactions with other organisms, including eukaryotic phytoplankton.

### Nucleotide sequence accession numbers.

The whole-genome sequence of *Sulfitobacter* sp. CB2047 was deposited in GenBank under the accession no. JPOY00000000. The version described in this paper is JPOY01000000, and consists of contig sequences JPOY01000001 to JPOY01000012.
